# Treatment of 28 patients with sclerosing hemangioma (SH) of the lung

**DOI:** 10.1186/1749-8090-7-34

**Published:** 2012-06-18

**Authors:** Yang Lei, Duan Yong, Ruan Jun-Zhong, Yang Zhi, Wang Zi-Tong

**Affiliations:** 1 Department of Thoracic of Beijing Chest Hospital, Beijing, China

**Keywords:** Lung benign tumor, Sclerosing hemangioma, Surgery

## Abstract

**Background:**

Sclerosing hemangioma (SH) of the lung is a kind of rare pulmonary tumor. Preoperative diagnosis of this tumor is difficult and it is now generally accepted that SH of the lung is benign lesions and surgical excision alone is curative. Herein, we present our experiences of treating 28 patients with SH.

**Methods:**

The medical records of 28 patients with SH from 1994 to 2010 at the Department of Thoracic Surgery in Beijing Chest Hospital were retrospectively reviewed.

**Results:**

There were 3 male and 25 female patients with sclerosing hemangioma and 50% of the patients were asymptomatic. Preoperatively, all the patients had undergone CT of chest and 5 patients had undergone PET scan but 4 patients were misdiagnosed as malignancy. There was no operative mortality or tumor recurrence despite that three different operative methods were undertaken.

**Conclusions:**

SH has a high incidence in middle-aged women. Most of SH is asymptomatic and the symptoms of SH are not related to the tumor size and distribution. The features of chest CT and PET are not specific. Bilateral or multiple lesions should not exclude the possibility of SH. Complete excision of lesion is a curable treatment method and there is no evidence to verify the need of adjuvant therapy.

## Background

Sclerosing hemangioma (SH) of the lung is a kind of rare pulmonary tumor which was first reported in 1956 by Liebow and Hubbel [1]. SH is composed of four histologic patterns including papillary, sclerotic, solid and hemorrhagic. At first the tumor was thought to be endothelial in origin, recently histochemical and ultrastructure studies suggested an epithelial (type II pneumocyte)origin [2]. Sclerosing hemangioma occurs in a wide age range (11-83 age)but is more common in middle-aged women [3,4] and has a higher incidence in Asia.

It is now generally accepted that sclerosing hemangioma of the lung is benign lesions and surgical excision alone is curative. This report describes our experience of surgery treatment of 28 patients with sclerosing hemangioma.

## Methods

From March 1994 to July 2010, 28 patients with sclerosing hemangioma underwent surgical treatment at the Division of Thoracic Surgery in Beijing Chest Hospital. Their medical records, operative procedures, histological examinations and follow-up data were reviewed. Before reviewed the data, we had obtained the approval of my institutional review board. The relation between the diameters of the tumors and the possibility of presenting symptoms was estimated using the Logistic regression by SPSS program, version 13.0. The frequency difference of presenting symptoms in the patients which the tumors located near the hilus of the lung and the other patients was estimated using the *χ*^2^ test(SPSS version 13.0).

## Results

### Clinical features

There were 3 male and 25 female patients with sclerosing hemangioma. The age at the time of diagnosis ranged from 25 to 58 years(mean age was 46.1 years). There were 14 patients who had no symptom and find the tumor on routine checkup. Among the symptomatic patients 7 presented with hemoptysis, 9 presented with cough, 3 presented with chest pain, 1 presented with expectoration sputum and 1 presented with fever.

We analyzed the diameters of the tumors and the possibility of presenting symptoms using the Logistic regression(SPSS version 13.0), the *P* value was 1.000 and suggested that the symptoms were not related to the tumor size. According to the location of the tumors, we divided the patients into two groups: central group(underwent lobectomy because of the near hilus location of the tumors) and peripheral group. The frequency of presenting symptoms showed no difference between the two groups (Table 1).

**Table 1:** The comparison of the frequencies of presenting symptom in the central group and peripheral group

	Without symptom	With symptoms	*χ*^2^	*P*
Central group	3	5	0.700	0.678
Peripheral group	11	9		

### Radiographic features

All but one patient had a solitary tumor on chest roentgenograms: 3 tumors located at left upper lobe, 9 tumors located at left lower lobe, 5 tumors located at right upper lobe, 4 tumors located at right middle lobe and 6 tumors located at right lower lobe. The exceptional patient had one major tumor which was 5.5 cm in diameter, located at left lower lobe and had multiple smaller tumors scattering at right lower lobe and left lower lobe.

Computer tomography (CT) scan of chest was performed in all the 28 patients. Solitary tumor was found in 27 patients. Among them malignancy was suspected in 3 patients for notch sign and spicular sign, the other patients were diagnosed as having benign lesions. In 1 patient, multiple tumors were found and diagnosed as metastases.

Positron emission tomography (PET) was performed in 5 patients. Among them, hyper metabolic lesions (SUV was 3.35 > the cut-off value 2.5) were found in the patient who had bilateral multiple tumors. There were no hypermetabolic lesions in PET of the other 4 patients.

### Operations

There were three types of operation methods we used. Enucleation was the most common procedure performed, 13 patients underwent the operation accounted for 46.4% of the patients. 7 patients underwent wedge resection accounted for 25% of the patients. We performed lobectomy in the other 8 patients(accounted for 28.6% of the patients) for the central locations of the tumors. One patient was diagnosed metastases because of the multiple tumors in bilateral lungs. This patient underwent video-assisted thoracoscopic surgery (VATS) wedge resection and obtain two tumors of the left lower lobe, both the tumors were diagnosed as sclerosing hemangioma (SH).

There were no operative mortality and morbidity. None of the patients underwent adjuvant therapy. During the follow-up ranging from 6 months to 16 years, none of the patients experienced tumor recurrence including the patients with multiple tumors.

## Discussion

Sclerosing hemangioma (SH) is more common in Asia and most patients with SH are women in the fifth decade. Some authors suggested that once a middle-aged Chinese women presents with a well-defined coin lesion on the chest film SH should be considered first [5]. In the current series, 89.3% of the patients were female and 34.6% of the patients aged from 40-49 ages. This data was compatible with most reports.

It is reported that SH of the lung were mainly found incidentally, over 70% of the patients were asymptomatic. The common symptoms of SH include hemoptysis, chronic cough and chest pain. In the current series, 50% of the patients presented some symptoms including hemoptysis, cough, chest pain, expectoration and fever. Some symptoms such as expectoration and fever were rarely seen in previously reports. The reason for more patients of our series presented symptoms than the other reports are not clear, we thought lack of regular body checkup could be related with the difference.

Some authors believed the symptoms were due to enlargement of the tumor and compression of surrounding tissue, and some other authors thought they were not related to tumor size or distribution [5-8]. To determine the relation between the symptoms and diameters of tumors, we analyzed the diameters of the tumors and the possibility of presenting symptoms using the Logistic regression(SPSS version 13.0)and found that the symptoms were not related to the tumor size. In our series 8 patients underwent lobectomy because the tumor located near to the hilus of the lung. So we divided all the patients into two groups: these 8 patients need lobectomy belonged to the central group and the other patients who underwent enucleation or wedge resection belonged to the peripheral group. To determine the relation between the locations and the symptoms, we estimated the frequency difference of presenting symptoms in the two groups using the *χ*^2^ test(SPSS version 13.0). We found that the frequencies of presenting symptoms did not differ between the two groups. Therefore we agreed that the symptoms of SH were not related to the tumor size and distribution.

On chest radiographs, SH is usually a peripheral, solitary nodule or mass with smooth margins. Calcification is seldom seen and cavitation does not occur. Some CT features of SH were demonstrated including inhomogeneous enhancement, the tail sign, the prominent pulmonary-artery sign, calcification, the air-trapping sign and so on, but none of them were specific for SH [9,10]^.^ Of all these 28 patients, most patients (20/28) presented a peripheral, solitary lesion with smooth margins on the chest radiographs and had one or more CT features as previously mentioned(inhomogeneous enhancement in 18 patients, the tail sign in 15 patients, the prominent pulmonary-artery sign in 10 patients, calcification in 3 patients and the air-trapping sign in 3 patients). But because of the poor specificity of the radiological features for SH, few of the patients were diagnosed as SH directly and most of the patients were diagnosed as benign lesions. So from previously reports as well as our experience, it is believed that most patients with SH could be diagnosed as benign lesions through the chest radiographs and CT but it is difficult to confirm the diagnosis as SH. Recently, fluorodeoxyglucose (FDG)-positron emission tomography (PET) is being used more and more to differentiate benign from malignant pulmonary lesions and it has been shown to be more efficacious than conventional chest CT. Since SH is thought to be a kind of benign tumor, the standard uptake value(SUV)of it should be less than 2.5 and then could be distinguished from lung cancer or other malignant tumors. But in fact, FDG is not a cancer-specific agent and some benign diseases including SH could show false-positive results on PET [11]. There have been some published reports about pulmonary SH with increased uptake on PET scan, and some authors thought the high uptake should be related to the tumor size or the potentially low-grade malignant nature of SH [12-15]. We performed PET scan on five patients, and found SUV of the patient who had bilateral multiple tumors and the largest tumor of the patient was 5.5 cm in diameter was greater than 2.5. Because of the small number of the patients undergone PET scan our experience of it was limited, but we thought the high uptake on PET scan cannot be used to exclude the diagnosis of SH and may be related to the tumor size or the potentially low-grade malignant nature of SH.

Traditionally, SH is a solitary lesion and thought to be benign. However, several reports about SH with lymph node metastasis, multiple and bilateral SH, recurrence of SH and SH with pulmonary metastasis have published in recent years [6,16-23]. These data suggested that SH may be potential malignant, but most authors believed that lymph node metastasis, multiple lesions, recurrence and pulmonary metastasis did not affect the prognosis. In the current series, chest CT of one patient showed bilateral multiple tumors in left and right lower lobe. Because of the multiple lesions the patient was diagnosed as metastasis. We performed VATS wedge resection on this patient and got two tumors of left lower lobe, both of them were diagnosed as SH. Thinking of the same nature of the other tumors, we did not resect them. After the operation, the patient did not receive any adjuvant therapy. Until now, 15 months later, the residual lesions have not changed. So we thought bilateral or multiple SH may be not related to the prognosis. But we have no experience of SH with lymph node or pulmonary metastasis and recurrence.

## Conclusion

Our data suggest that SH has a high incidence in middle-aged women. Most of SH is asymptomatic and the symptoms of SH are not related to the tumor size and distribution. Chest CT and PET can offer some information to diagnose accurately but the features are not specific. Bilateral or multiple lesions should not exclude the possibility of SH. Complete excision of lesion is a curable treatment method and there is no evidence to verify the need of adjuvant therapy.

## Abbreviations

SH = Sclerosing hemangioma; PET = Positron emission tomography; SUV = Standard uptake value.

## Competing interests

Yang Lei and other co-authors have no competing interest.

## Authors’ contributions

YL is the author of the paper. DY helped in the data and literature research. RJ-Z checked the paper and performed literature research. YZ checked the paper and performed linguistic control. WZ-T was head of the department and supervised the study. All authors read and approved the final manuscript.
